# Estimation of the Shear Viscosity of Mixed-Polymer Materials for Screw Extrusion-Based Recycling Process Modeling

**DOI:** 10.3390/polym16101339

**Published:** 2024-05-09

**Authors:** Christian Kneidinger, Emil Wagner, Manuel Längauer, Gernot Zitzenbacher

**Affiliations:** Department of Materials Technology, School of Engineering, University of Applied Sciences Upper Austria, Stelzhamerstr. 23, 4600 Wels, Austria; emil.wagner@fh-wels.at (E.W.); g.zitzenbacher@fh-wels.at (G.Z.)

**Keywords:** polymer recycling, predictive modeling, rheological properties, extrusion, polypropylene, polyamide 12, capillary rheometry, blends

## Abstract

The scope of this work is the development of a method to estimate the temperature and shear rate-dependent viscosity of mixtures composed of two polymers. The viscosity curve of polymer mixtures is crucial for the modeling and optimization of extrusion-based recycling, which is the most efficient way to recycle polymeric materials. The modeling and simulation of screw extruders requires detailed knowledge of the properties of the processed material, such as the thermodynamic properties, the density, and the rheological behavior. These properties are widely known for pure materials; however, the incorporation of impurities, like other polymers in recycled materials, alters the properties. In this work, miscible, immiscible, and compatibilized immiscible polymer mixtures are considered. A new method based on shear stress is proposed and compared to the shear rate-based method. Several mixing rules are evaluated for their accuracy in predicting mixture viscosity. The developed methods allow the prediction of the viscosity of a compatibilized immiscible mixture with deviations below 5% and that of miscible polymer mixtures with deviations below 3.5%.

## 1. Introduction

The recycling of polymer films is not only ethically required but also enforced by the European Union in its Directive 94/62/EC, which was first formulated in 1994 and revised in 2018. It states that by end of December 2025, 50 wt% of packaging plastics must be recycled. This is raised to 55% by the end of 2030 [1]. Plastic films are produced by either flat-film or blown-film extrusion processes. In these processes, single-screw extruders are commonly used. From a hopper polymer, bulk material is fed to the solid conveying zone of the single-screw extruder. Afterwards, the bulk material is compacted, compressed, plasticated, conveyed, mixed, and extruded through a die to receive the characteristic film shape [2,3,4,5,6].

The packaging of food and other perishable goods demands certain barrier properties from the packaging materials [7]. Mostly, oxygen (O_2_) and water vapor (H_2_O) are unwanted in the products, whereas carbon dioxide (CO_2_) is often used as an inert gas inside the packaging. The diffusion of gases through porous solids can be described with Knudsen diffusion [8,9,10]. The physical resistance that opposes the diffusivity is the barrier property [11]. The permeation process can be explained by a combination of Henry’s and Fick’s laws [12]. Since different polymers exhibit different barrier properties, a combination of different layers of polymers can suffice to reach the imposed limits of permeability.

These combinations are called multilayer films, which are usually created by using multiple extruders that are connected to a feedblock that arranges the layers and feeds them to a die. One of the major challenges that comes at the end of life of multilayer film packaging is the recycling process. Films can be dumped in landfills, energy recovery can be employed, or they can be recycled [13]. The most common choice of use at the end of life in Europe is still energy recovery or thermal recycling [14]. Other routes are chemical recycling, feedstock recycling, or mechanical recycling. The enforcement of directives and regulations will tip the balance towards mechanical recycling, in which end-of-life products are collected, sorted, and reprocessed to new products. Contrary to chemical recycling [15], mechanical recycling does not allow the separation of different polymers of multilayer films. Those polymers are processed in the same material stream. The mixing of polymers has effects on most material properties, especially the rheological, mechanical, and thermal properties. Compatibilization of immiscible polymers can assist in hindering the deterioration of these properties [16,17].

Kallel et al. [18] discussed the effect of compatibilization of polyethylene–polystyrene (PE–PS) and polyethylene–polypropylene (PE–PP) blends. By adding maleic acid anhydride-grafted ethylene–propylene copolymer (EP-g-MAH) for PE–PP and styrene–ethylene–butadiene–styrene (SEBS) for PE–PS, respectively. They analyzed rheological behavior with a parallel-plate rheometer and concluded that at low shear rates, compatibilizers act as emulsifiers in the molten state. This effect is diminished at high shear rates, where curves tend to converge. This effect was also previously reported by Palierne [19] and Bousmina [20]. Yoo et al. [21] showed the effect of maleic acid anhydride-grafted styrene–acrylonitrile (SAN-g-MAH) on the rheological properties of polypropylene carbonate–polymethyl methacrylate (PPC–PMMA) blends and reported a strong enhancement effect of the compatibilization, even at parallel-plate rheometry testing frequencies of 10^2^ rad·s^−1^, corresponding to a maximum shear rate of approximately 400 s^−1^. Kozlowski and Mantia [22] reported a quite different effect when studying blends of PP and LCP with and without the addition of PPgMAH (maleic acid anhydride-grafted polypropylene). The shear viscosities derived from capillary rheometry showed a decrease with the addition of the compatibilizer. Holsti-Miettinen et al. [23] reviewed the effect of the chemical structure of compatibilizers on the properties of compatibilized PP–polyamide 6 (PA 6) blends. They used EBA-g-FA (fumaric acid-grafted ethylene–butylene acrylate), PPgMAH, SEBS-g-MAH and E-AE-GMA (ethylene–ethyl acrylate–glycidyl methacrylate terpolymer with glycidyl methacrylate) and tested the blends using a capillary rheometer. In a shear rate region between 10^2^ and 10^4^ s^−1^, all the compatibilizer additions led to an increase in viscosity of the blend. SEBS-g-MAH had the strongest effect in this study, revealing even higher viscosities than the single components at low shear rates.

The calculation of extrusion characteristics demands rheological material data [3,4,5,6]. By altering this behavior through the incorporation of impurities like other polymers, the process design must be adapted [6,24,25,26]. Since it would be far too time-consuming to test every material mixture before extrusion, material models for mixtures have been developed and are now more important than ever before. Although several mixing rules exist, there is no calculation method that considers the shear rate/shear stress-dependent behavior or polymers, until now.

Bingham [27] (1914) and Heitmiller et al. [28] (1964) formulated an approach with a reciprocal additive relation to calculate the shear viscosity of a mixture of two polymers considering Newtonian fluids with the same density. An expression for the mixture’s viscosity $\eta_{M_{B/H}}$ is derived by balancing the flow rates and formulated as:

(1) $$\eta_{M_{B/H}}=\frac{1}{\frac{v_{A}}{\eta_{A}}+\frac{1-v_{A}}{\eta_{B}}}$$

where $v_{A}$ is the volume fraction of component A, and $\eta_{A}$ and $\eta_{B}$ are the shear viscosities of component *A* and *B*, respectively. Heitmiller et al. [28] assume that the liquids are divided into many layers that are flowing adjacently without mixing. Lin [29] (1979) presented a comparable approach with an additional interaction parameter that considers the interfacial slip between the layers [30,31,32,33] or instabilities [34,35,36] that may influence the results.

Tsenoglou [37] (1987) estimated the stress relaxation modulus of entangled polymer blends and formulated his mixing rule for a mixture’s zero-shear viscosity $\eta_{0,M_{T,Orig}}$:

(2) $$\eta_{0,M_{T,Orig}}=v_{A}^{2}\eta_{0A}+4v_{A}v_{B}\left(\frac{\eta_{0A}\eta_{0B}}{\eta_{0A}+\eta_{0B}}\right)+v_{B}^{2}\eta_{0B}$$

where $v_{B}$ is the volume fraction of component *B.* As we deal with mixtures of two materials, $v_{B}$ is 1 minus $v_{A}$. $\eta_{0A}$ and $\eta_{0B}$ are the zero-shear viscosities of component *A* and *B*, respectively. The mixing rule of Tsenoglou was adapted as described in [38] to analyze if it allows to estimate the shear rate-dependent viscosity $\eta_{M_{T}}$ of a mixture of polymers in a wide range of shear rates:

(3) $$\eta_{M_{T}}=v_{A}^{2}\eta_{A}+4v_{A}v_{B}\left(\frac{\eta_{A}\eta_{B}}{\eta_{A}+\eta_{B}}\right)+v_{B}^{2}\eta_{B}$$

Arrhenius [39] (1887) and Bersted et al. [40] (1981) proposed a mixing rule $\eta_{M_{A/B}}$ for blends of branched and linear components, which is then assumed to be independent of the shear rate. The viscosity of the mixture is given as:

(4) $$\eta_{M_{A/B}}=\eta_{A}^{w_{A}}\eta_{B}^{w_{B}}$$

where $w_{A}$ and $w_{B}$ are the weight fractions of components *A* and *B*. The model is validated with viscosity data of a mixture of high- and low-density polyethylene (HDPE and LDPE) from another study [41]. Furthermore, the model is used to describe the viscosity of neat HDPE as a mixture as well.

Friedman and Porter [41] published a mixing rule that originates in the work of Kendall and Monroe [42]. Their mixing rule for predicting the viscosity of a mixture $\eta_{M_{KM/FP}}$:

(5) $$\eta_{M_{KM/FP}}={\left(w_{A}\eta_{A}^{\frac{1}{c}}+w_{B}\eta_{B}^{\frac{1}{c}}\right)}^{c}$$

uses a scaling exponent c between the viscosity and the molecular weight, which is usually around 3.4 [43,44]. The simplest mixing model is a linear model $\eta_{M_{lin}}$:

(6) $$\eta_{M_{lin}}=\eta_{A}w_{A}+\eta_{B}w_{B}$$

Walther [45] presented his double logarithmic mixing rule for predicting the viscosity of a mixture $\eta_{M_{W}}$ in 1931. According to Gao and Li [46], the constant C is set to 0:

(7) $$\ln\ln\left(\eta_{M_{W}}+C\right)=w_{A}\ln\ln\left(\eta_{A}+C\right)+w_{B}\ln\ln\left(\eta_{B}+C\right).$$

Cragoe [47] presented his reciprocal logarithmic mixing rule for predicting the viscosity of a mixture $\eta_{M_{Cr}}$ in 1933. According to Gao and Li [46], the constant L is set to 2000:

(8) $$\frac{1}{\ln\left(L\;\eta_{M_{Cr}}\right)}=\frac{w_{A}}{\ln\left(L\;\eta_{A}\right)}+\frac{w_{B}}{\ln\left(L\;\eta_{B}\right)}$$

When one of these mixing rules is used to predict the viscosity of polymer mixtures, the shear rate/shear stress-dependent viscosity of the pure materials must be considered. From the mentioned mixing rules, the rule of Bersted et al. [40] is the only one that was applied considering the shear rate. Contrary, Utracki and Wilkie [48] recommend estimating the viscosity of a mixture based on shear stress, but they do not show a method of how to do this in detail. In theory, a shear stress-based evaluation method should allow an estimation of the rheological behavior of blends with higher accuracy.

This paper presents a method to predict the shear rate/shear stress-dependent viscosity of a mixture of two polymers from the viscosity of the pure polymers and from the volume fraction. A new method to calculate the viscosity of a polymer mixture based on shear stress considering several mixing rules is proposed. The results are compared to measured values, which are also compared to a shear rate-dependent calculation method that is also considering the previously mentioned mixing rules. Accurately predicting the mixture viscosity can drastically speed up the determination of the material behavior for the simulation of polymer recycling processes. Moreover, the effect of compatibilization on the viscosity of PP–PA12 mixtures is analyzed.

## 2. Materials and Methods

The polymeric materials used are PP HB600TF and PP HD234CF (both Borealis AG, Vienna, Austria) and polyamide 12 (PA12) Grilamid L25 (EMS-CHEMIE HOLDING AG, Herrliberg, Switzerland). Both PP grades are typical film extrusion materials. Maleic acid anhydride-grafted PP (PPgMAH) Orevac CA 100 (Arkema Functional Polyolefines, Colombes Cedex, France) was used as a coupling agent between the PP HB600TF and the PA12 grade. The polymeric materials with the corresponding melt flow rate (MFR) or melt volume rate (MVR) and the recommended processing temperatures are given in Table 1.

**Figure 1:**
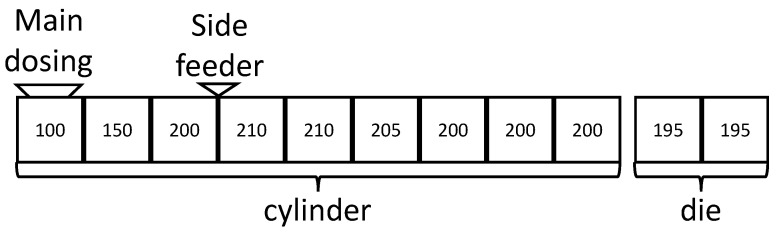
Setup of the twin-screw extruder with the temperature profile used (all values in °C) along the cylinder and the die and the position of the dosing systems.

The polymer mixtures were produced using a co-rotating twin-screw extruder: Leistritz ZSE 27 Maxx (Leistritz Group, Nuremberg, Germany). The temperature profile used was held constant for all material blends (see Figure 1) and a standard screw configuration was used.

The contaminating polymer was supplied by the side-feeder (Congrav OP5 dosing, Kubota Brabender Technologie GmbH, Duisburg, Germany) in each setup, whereas the base polymer was provided by the main dosing (Congrav M, Kubota Brabender Technologie GmbH, Duisburg, Germany). As a coupling agent, the PPgMAH masterbatch was added to the PP_1_ in a preceding compounding process.

The amount of coupling agent that was needed to obtain a satisfying adhesion between PP and PA12 was determined in a previous step. For this purpose, the PP base polymer PP_1_ was blended with 1, 2, 3, 4 and 5 wt% of the PPgMAH masterbatch. Afterwards, 20 wt% PA12 was added to those PP compounds with different amounts of coupling agent, resulting in the mixtures that are shown in Table 2.

The test specimens for the Charpy impact test in Table 2 were produced as prescribed by DIN EN ISO 20753:2019 [49] using an injection molding machine Engel Victory 330/80 Tech (Engel Austria GmbH, Schwertberg, Austria). The process settings were a melt temperature of 230 °C, a mold temperature of 40 °C, pressure holding time of 40 s and a cooling time of 13 s. The middle part of the multipurpose test specimen was separated with a custom-made cutter to be used for Charpy impact testing, following the DIN EN ISO 179 norm [50]. The samples were conditioned at a temperature of 23 °C at 50% relative humidity for a duration of 88 h in a CLS climatic chamber 70/600 (Klima Temperatur Systeme GmbH, Jennersdorf, Austria) before conducting the impact tests using a Zwick 5113E pendulum impact tester (ZwickRoell GmbH & Co. KG, Ulm, Germany) equipped with a 50 J pendulum. This mechanical test was repeated five times for each blend. After deciding on which amount of coupling agent to use for the main set of rheological experiments, those blends were manufactured as well. Table 3 shows all the different blends used in the rheological experiments.

To determine the morphology of the blends, field electron scanning electron microscopy (FE-SEM) was deployed. Pellets of the blends were submerged in liquid nitrogen, placed in a cotton cloth, and broken with a hammer. The resulting pieces were placed on a sample holder and sputtered with a thin layer of gold before analyzing them with a MIRA3 LHM FE-SEM (Tescan Orsay Holding, Brno, Czech Republic).

The rheological tests were performed using a high-pressure capillary rheometer Rheograph 6000 (Göttfert, Buchen, Germany) with the corresponding Labrheo software (version 4.3.3). The tests were conducted at a temperature of 230 and 250 °C deploying capillaries with a diameter of 1 mm and an L/D ratio of 5, 10, 20 and 30, respectively.

Depending on the L/D ratio of the capillary dies in use, one pressure transducer was used to detect the chamber pressure before the inlet of the capillary. The measured pressure loss was corrected using Bagley correction [51]. The true shear viscosity curve was obtained employing Weissenberg–Rabinowitsch correction [52]. The resulting true viscosity curves were fitted using a simplified Bird–CarreauQ –Yasuda (BCY) [53] model (Equation (9)) in combination with the least-square method and a numerical solver:

(9) $$\eta_{Carreau-Yasuda}=\left(\eta_{0}\right){\left[1+{\left(B\dot{\gamma }\right)}^{a}\right]}^{\frac{n-1}{a}}$$

where $\eta_{0}$ is the zero-shear viscosity, B is a time constant describing the reciprocal value of the shear rate where the viscosity changes from the Newtonian zero-shear rate plateau to shear thinning behavior, n is the power-law exponent and a is a material specific model parameter that describes the transition from the Newtonian zero-shear-rate plateau to shear thinning behavior in more detail.

Bersted et al. [40] presented a shear rate-based method of applying their mixing rule, but they did not describe the method how to do this in detail. We propose performing the shear rate-based method in the following manner. The first step is to fit a viscosity model (e.g., that of BCY [53]) for the pure materials. Four additional evaluation steps are needed to predict the viscosity of a mixture as a function of the shear rate assuming that the viscosity models of the pure materials are known:

1. Define several shear rate values for the calculation.
2. Calculate the viscosity values of the pure polymers at the defined shear rate values.
3. Calculate the viscosity of the mixture using one of the previously mentioned mixing rules (linear mixing rule, Kendall and Monroe [42]/Friedman and Porter [41], Tsenoglou [37], Arrhenius [39]–Bersted et al. [40], Bingham [27]–Heitmiller et al. [28]).
4. If needed, apply a viscosity model on the calculated viscosity of the mixture.

## 3. Results

### 3.1. Shear Stress-Based Calculation of the Mixture Viscosity

Utracki and Wilkie [48] recommend the calculation of the viscosity of polymer mixtures based on shear stress, because higher accuracy is expected. The shear stress-based calculation method is more complicated than the shear rate-dependent calculation, as numerical calculations are needed. The following evaluation steps must be carried out after fitting a viscosity model (e.g., that of BCY [53]) for the pure materials.

1. In the first step, several shear stress values for the calculation are defined. In this paper, the shear stress values of the mixtures from the rheological tests were used.
2. The viscosity values of the pure polymers are calculated for those defined shear stresses. For this purpose, the basic relation between shear stress and shear rate is used. The shear rate value $\dot{\gamma }$ must be adapted until the calculated shear stress value equals the shear stress value that was defined in step 1. This can be done with a numerical solver, as presented in [6,54,55]
3. After that, the mixing rules presented in the Introduction (linear mixing rule, Kendall and Monroe [42]/Friedman and Porter [41], Tsenoglou [37], Arrhenius [39]–Bersted et al. [40], Bingham [27]–Heitmiller et al. [28]) are employed to calculate the shear viscosity of the mixture $\eta_{m}$ as a function of the shear stress τ. The comparison of the results using the mixing models to the measured values is much more complicated for the shear stress-dependent calculation, as the shear rate must be calculated from the shear stress and the shear viscosity and deviates from the measured values. Thus, the next step is needed.
4. The shear rate ${\dot{\gamma }}_{M,Model}$ can now be calculated from the viscosity of the mixture $\eta_{M,Model}$ and from the shear stress τ:
  (10) $${\dot{\gamma }}_{M,Model}=\frac{\tau }{\eta_{M,Model}}$$
  The results of this calculation are several points that describe the viscosity of a mixture and the corresponding shear rate values for the different mixing rules.
5. Then, a viscosity model is used to approximate the calculated viscosity values of the mixture $\eta_{M,Model}$ considering the shear stress values obtained in step one and the shear rate value ${\dot{\gamma }}_{M,Model}$ calculated in step four. In this paper, the simplified BCY (see Equation (9)) was used.
6. To compare the results of the shear stress-dependent calculation, the viscosity models from step five must now be applied to calculate the viscosity of the mixture for the different models at the shear rates from the rheological tests.

The deviations of the mixture viscosity that are presented in this paper are calculated as the difference between the predicted model values and the values received from the rheological tests. A positive deviation shows an overestimation by the mixing rules and a negative deviation and underestimation of the predicted mixture viscosity.

### 3.2. Comparison of the Mixing Models

**Figure 2:**
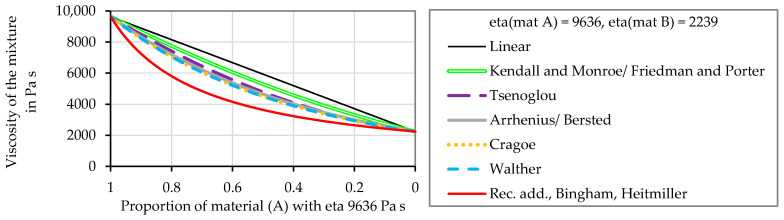
Exemplary evaluation of the predicted mixture viscosity using different mixing models, assuming two polymeric materials without considering shear rate-dependent behavior. Calculations according to Equations (1) and (3)–(8). The viscosity of material A is 9636 Pa s and that of material B is 2239 Pa s.

The different mixing rules from the literature are first compared against each other. A test evaluation for two materials with the viscosities 2239 and 9636 Pa s was performed to compare the predicted mixture viscosity values obtained with the different mixing models. As Figure 2 shows, the linear mixing rule predicts the highest mixture viscosity. The mixing rules of Kendall and Monroe [42] and Friedman and Porter [41] deliver viscosity values that are slightly below the values calculated with the linear model. The values calculated with Tsenoglous’s [37] mixing rule are lower than those predicted according to Kendall and Monroe [42]/Friedman and Porter [41], and Arrhenius [39] and Bersted et al. [40]. The viscosity values obtained employing the mixing rules of Cragoe [47] and Walther [45] show nearly the same behavior as those obtained according to Arrhenius [39]–Bersted et al. [40]. Consequently, the mixing rule of Cragoe and Walther was not further considered. When applying the reciprocal additive relations of Bingham [27] and Heitmiller et al. [28], the lowest viscosity values were obtained.

### 3.3. Shear Viscosity of PP Contaminated with a Different PP Grade

**Figure 3:**
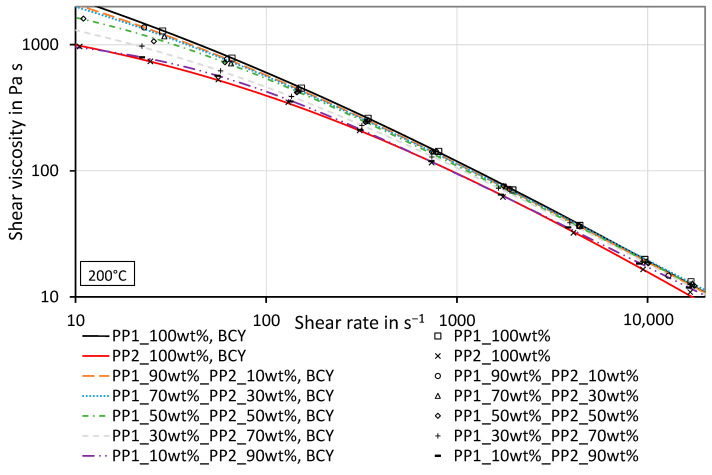
Shear viscosity curves of mixtures composed of PP_1_ and PP_2_ with different concentrations and shear viscosity curves of the pure polymers including the BCY fits at a testing temperature of 200 °C.

An overview of the shear viscosity curves for the different mixtures of PP_1_ and PP_2_ is given in Figure 3. The lines represent the BCY fits of the measured values, and the measured values are shown as symbols. Nearly all measured values fall between the viscosities of the pure materials. The corresponding parameters of the fits can be seen in Table A1 in Appendix A. The measurement data can be found in Appendix A in Table A2 and Table A3. All presented viscosity and shear rate values are so-called true values.

**Figure 4:**
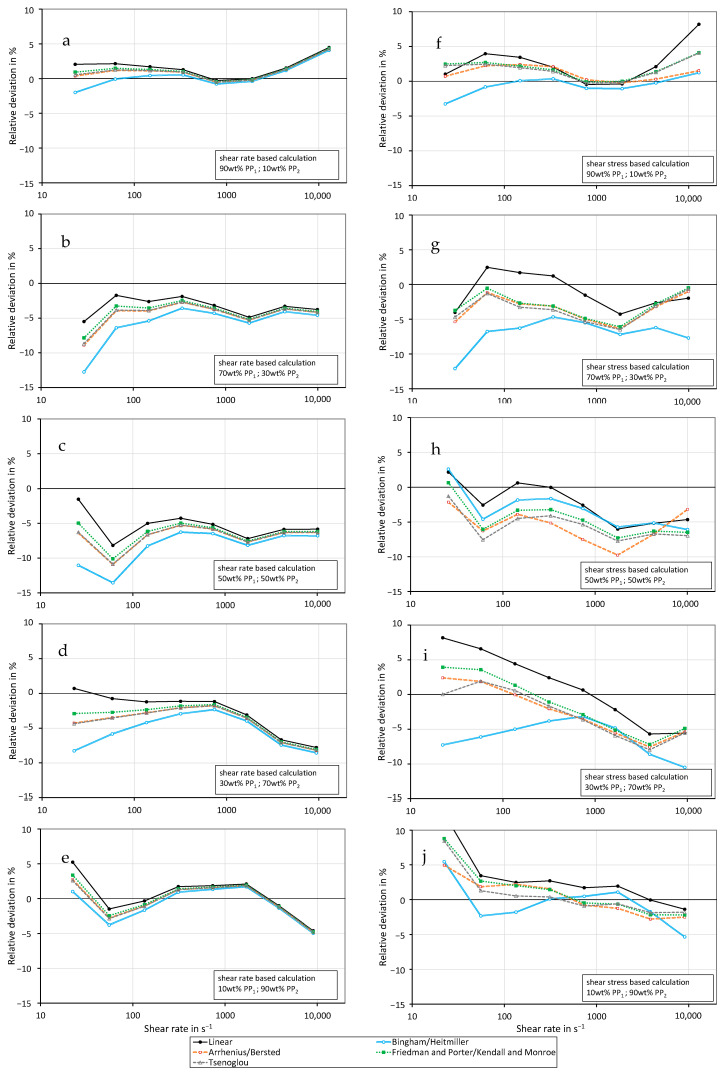
Relative deviation of the shear viscosity of PP_1_ mixed with different concentrations of PP_2_ calculated with the mixing models based on shear rate (**a**–**e**) or shear stress (**f**–**j**) from the measured values at a temperature of 200 °C. (**a**,**f**) 10 wt% PP2, (**b**,**g**) 30 wt% PP2, (**c**,**h**) 50 wt% PP2, (**d**,**i**) 70 wt% PP2, (**e**,**j**) 90 wt% PP2.

Figure 4 shows the relative deviation between the predicted and the measured shear viscosity of polypropylene PP1 mixed with different concentrations of PP2. The results of the shear rate-based calculation method are depicted on the left-hand side in charts a–e. The deviations obtained by employing the shear stress-based calculation method are presented on the right-hand side in charts f–j. For the prediction of mixture viscosity, the linear mixing rule, the rule of Bingham and Heitmiller, the rule of Arrhenius and Bersted, the rule of Kendall and Monroe/Friedman and Porter, and the rule of Tsenoglou are used. The mixtures with 10 wt% PP_2_ and with 90 wt% PP_2_ are described very well with all mixing models and both calculation methods. In the case of the other mixtures, the shear rate-based calculation method delivers negative deviations for nearly all measurement points. In this case, the linear model predicts the mixture viscosity best, which is demonstrated by the lowest deviations from the measured values. The shear stress-dependent calculation is more accurate for the mixtures with 30, 50, and 70 wt% PP_2_, which can be seen in the lower deviations from the measured values.

It seems like low concentrations of another polymer (10%) lead to rather strong deviations that can be described well with the reciprocal additivity relationship of Bingham and Heitmiller et al. [28]. Mixtures with a higher concentration of impurities lead to comparatively higher viscosities and can be described by the linear mixing rule in the best manner. This holds true for both the shear rate-based and the shear stress-based calculation methods. For the shear rate-based calculation, the measured values are even higher than the linear mixing rule predicts.

### 3.4. Shear Viscosity of PP Contaminated with PA12

**Figure 5:**
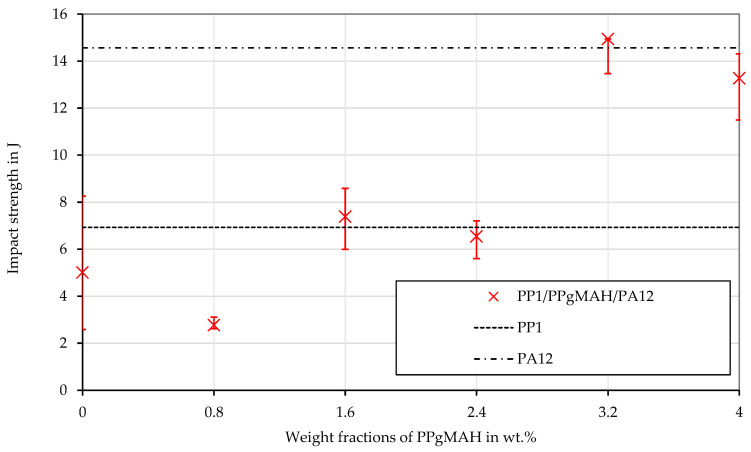
Impact strength of the PP_1_–PA12 specimen with different concentration of the coupling agent PPgMAH and of the pure material PP_1_. The concentration of PA12 is always 20%. The signs show the median values of six measurements, the error bars show the maximum and the minimum value. The impact strength of the pure PP_1_ and the pure PA12 are shown as horizontal lines.

Figure 5 shows the results of the impact tests of the PP compounds with different PPgMAH concentrations. PP_1_ has approximately half the impact strength of PA12 (the values are presented as lines). Compounding PP_1_ with 20 wt% PA12 without compatibilization leads to a decrease in the impact strength. Adding 1, 2 or 3 wt% PPgMAH to PP_1_ before blending it with PA12, which results in 0.8, 1.6 or 2.4 wt% PPgMAH in the final mixture, has only minor effects on the impact strength. At a concentration of 3.2 wt% (4 wt% PPgMAH in PP_1_), a sudden increase occurs, and the impact strength jumps to those of neat PA12. At a PPgMAH content of 4 wt% (5 wt% PPgMAH in PP_1_), no further enhancement of the impact strength can be found, which is why a PPgMAH content of 4% was chosen for the rheological tests. Due to the enhanced impact strength, it is assumed that sufficient compatibilization between PP_1_ and PA12 is obtained at this concentration of coupling agent.

**Figure 6:**
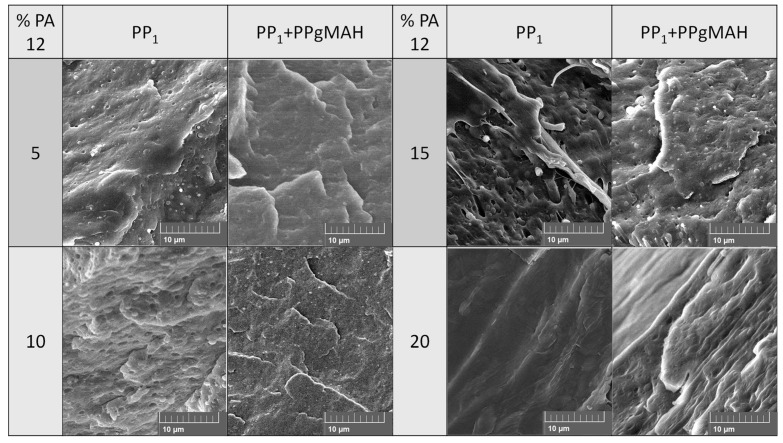
SEM images of cryogenically broken pellets of PP_1_–PA12 with and without coupling agent for different PA12 concentrations.

Figure 6 shows SEM-images with a magnification of 5000 of cryogenically broken pellets of blends of PP_1_–PA12 with and without coupling agent and a different PA12 concentration. Especially at lower PA12 concentration, the effect of the compatibilizer well visible. Except for a concentration of 20 wt% PA12, the blends without coupling agent appear as one large phase with small, bubble-shaped inclusions. Those inclusions become larger and more numerous with higher PA12 concentrations. In the SEM images of the samples with compatibilization, the inclusions appear much smaller and better dispersed. At a PA12 concentration of 20 wt%, the phases are not easily distinguishable anymore.

**Figure 7:**
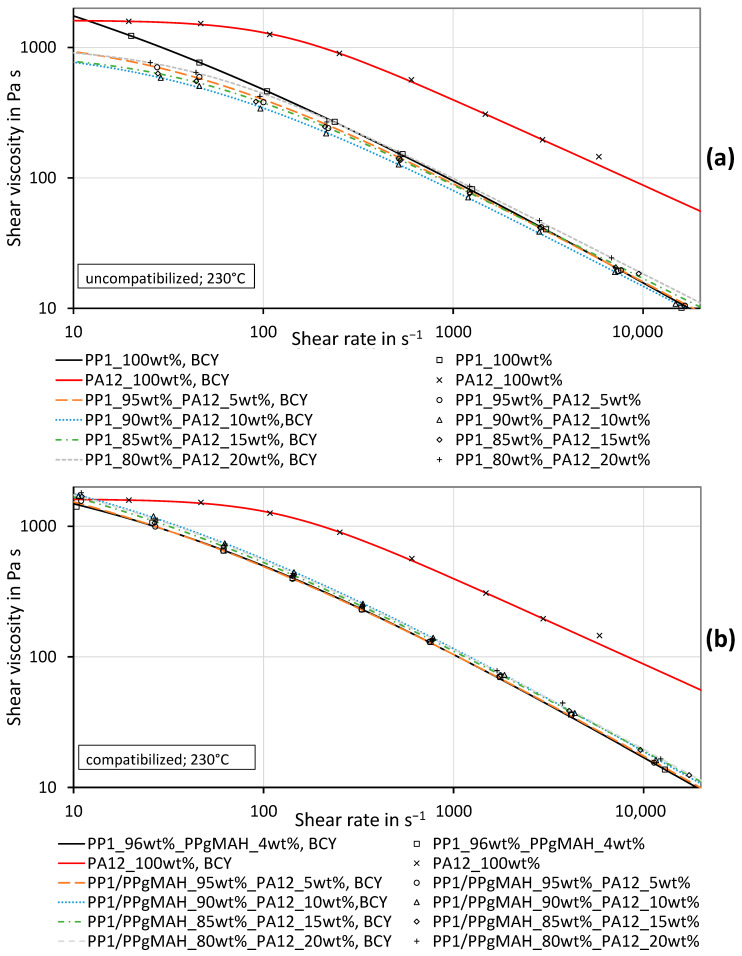
Shear viscosity curves of the PP_1_–PA12 blends and the pure materials including the BCY fits of uncompatibilized (**a**) and compatibilized (**b**) PP_1_ with different concentrations of PA12 at a testing temperature of 230 °C.

**Figure 8:**
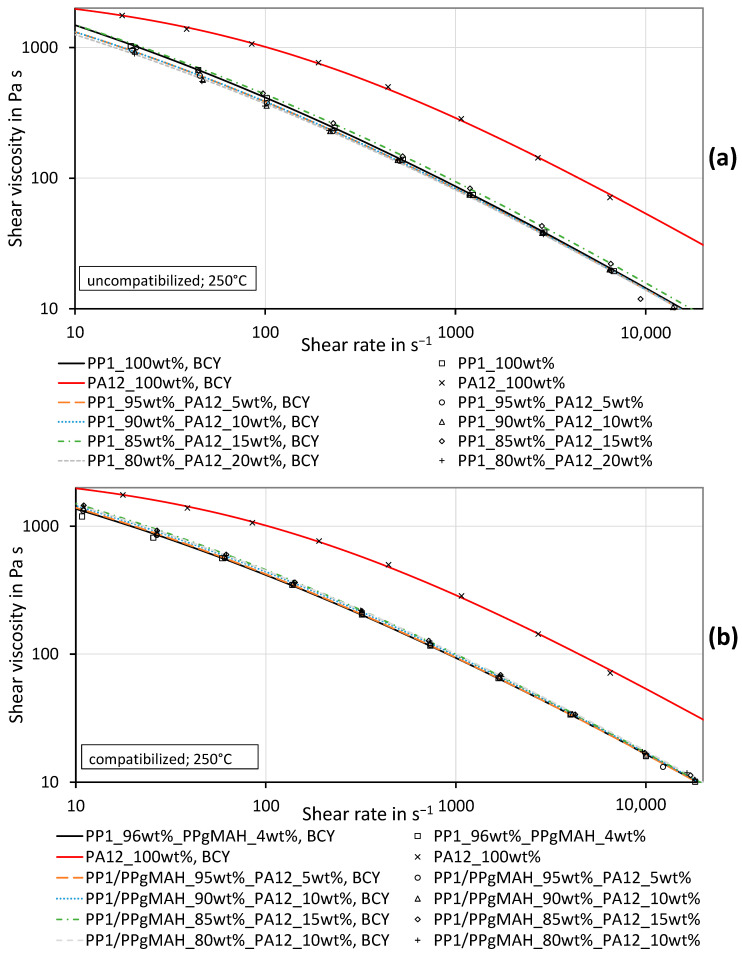
Shear viscosity curves of the PP_1_–PA12 blends and the pure materials including the BCY fits of uncompatibilized (**a**) and compatibilized (**b**) PP_1_ with different concentrations of PA12 at a testing temperature of 250 °C.

Figure 7 and Figure 8 show the measured shear viscosity curves of the uncompatibilized PP_1_–PA12 blends, the compatibilized PP_1_–PA12–PPgMAH blends and the pure materials at temperatures of 230 °C and 250 °C. Especially at higher shear rates, PA12 exhibits a far higher shear viscosity than PP_1_. The viscosity of the uncompatibilized blends does not systematically rise with increasing PA12 content. Especially at a temperature of 230 °C, the viscosity of the blend at lower shear rates is even lower than the viscosity of pure PP_1_. At higher shear rates, the blends have a viscosity that is slightly higher than or similar to the viscosity of pure PP_1._ The standard deviations of the measurements are very low and therefore are not shown in the charts.

The blends with PP_1_ with coupling agent show a different behavior. The measured viscosities of those blends are slightly higher than the viscosity of pure PP_1_ along the total shear rate range. The values of the mixtures are slightly above the values of pure PP_1_. This is not astonishing, as the highest concentration of PA12 is only 20 wt%. The parameters obtained by the mathematical fit of the curves, as well as their deviation from the measured data, are depicted in Table A4 in Appendix B. The measurement data can be found in Appendix B in Table A5 and Table A6. All presented viscosity and shear rate values are so-called true values.

**Figure 9:**
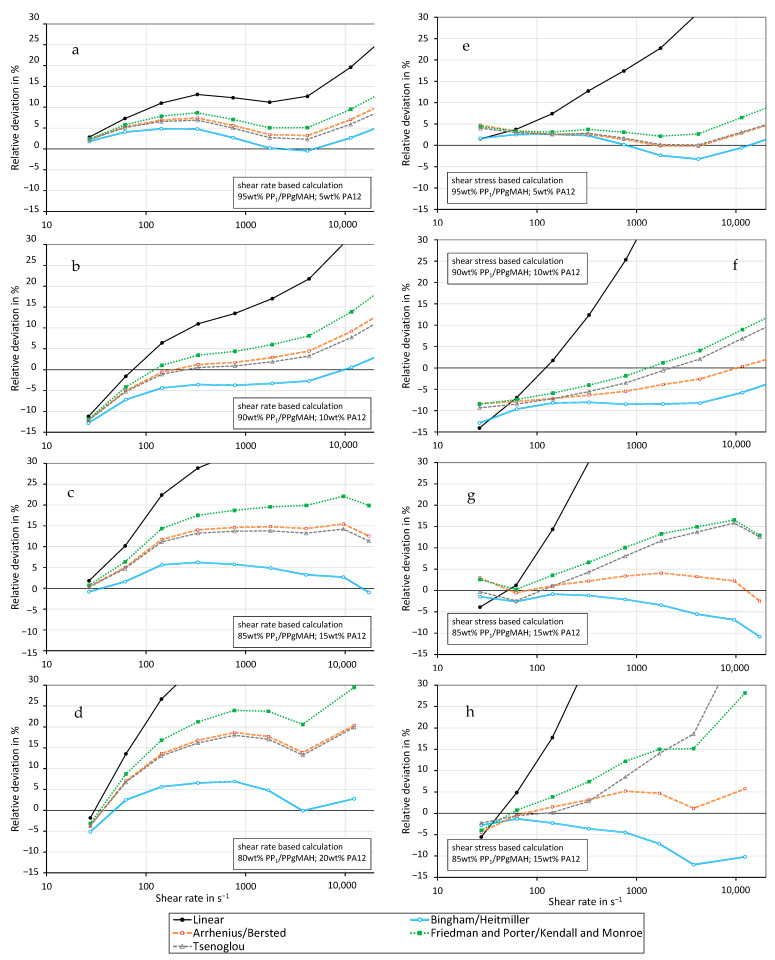
Relative deviation of the predicted shear viscosity from the measured values for different mixing models using the shear rate-based (**a**–**d**) and shear stress-based (**e**–**h**) calculation method for PP1–PA12 blends with coupling agent at a temperature of 230 °C. ((**a**,**e**) 5 wt% PA12, (**b**,**f**) 10 wt% PA12, (**c**,**g**) 15 wt% PA12, (**d**,**h**) 20 wt% PA12).

**Figure 10:**
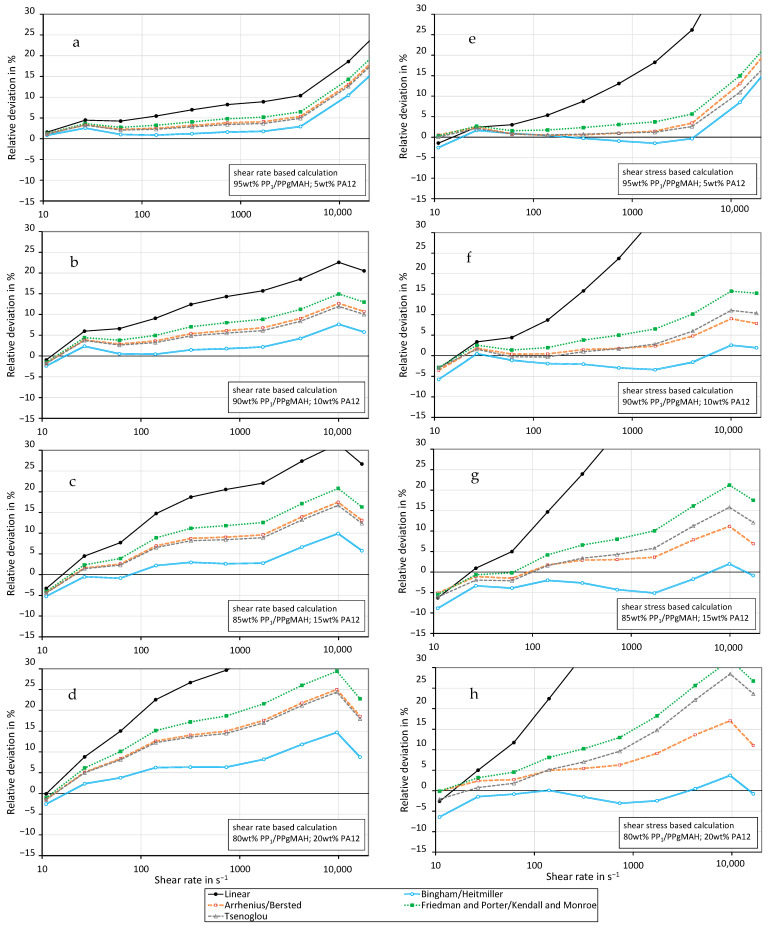
Relative deviation of the predicted shear viscosity from the measured values for different mixing models using the shear rate-based (**a**–**d**) and shear stress-based (**e**–**h**) calculation method for PP_1_–PA12 blends with coupling agent at a temperature of 250 °C. ((**a**,**e**) 5 wt% PA12, (**b**,**f**) 10 wt% PA12, (**c**,**g**) 15 wt% PA12, (**d**,**h**) 20 wt% PA12).

The previously described mixing rules were applied to the compatibilized immiscible mixtures in a fashion similar to the miscible ones. Figure 9 and Figure 10 show the relative deviation of the mixing models. The mixing models were calculated using the measured values depicted in Figure 7 and Figure 8. The graphs show that the Bingham–Heitmiller model yields the least relative deviation compared to the other mixing rules when using the shear rate-based calculation method. This holds true for all mixtures at both 230 °C and 250 °C. The Bingham–Heitmiller model also deviates the least at 250 °C when calculated through the shear stress-based method. At 230 °C, the Arrhenius–Bersted model is more accurate. In contrast to the miscible mixtures, the linear model results in the highest calculated relative deviation for the immiscible blends. Overall, the relative deviations are higher than those found in blends made from the two PP grades.

## 4. Discussion

**Figure 11:**
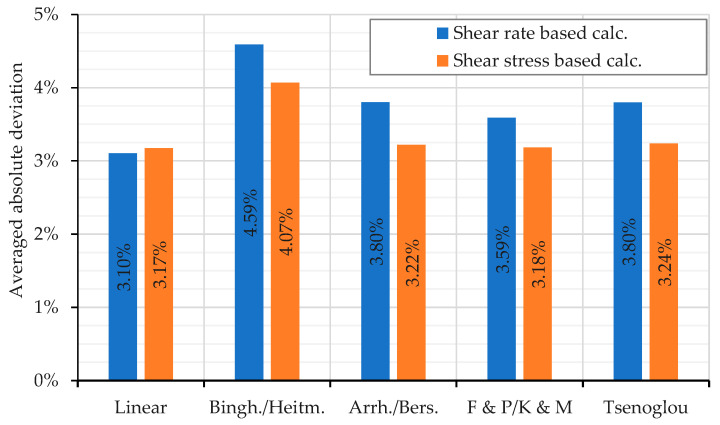
Averaged absolute relative deviation over the total shear rate or shear stress range for the different mixing models applied to mixtures of PP_1_ with all concentrations of PP_2_. Averaged absolute values of the deviations presented in Figure 4.

**Figure 12:**
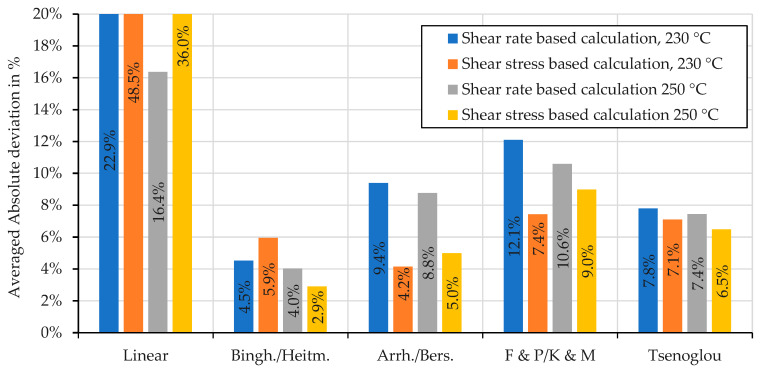
Averaged absolute relative deviations of the mixing models applied to compatibilized PP_1_–PA12 blends at 230 °C and at 250 °C. Averaged absolute values of the relative deviations presented in Figure 9 and Figure 10.

To obtain a better overview of the results, the mean absolute deviations of the individual mixing rules were determined for both the shear rate-based and the shear stress-based calculation method. The results are shown in Figure 11 and Figure 12. In general, using the shear stress-based calculation method predicts more accurate mixture viscosities for nearly all employed mixing rules, which is demonstrated by the low deviations. Exceptions are the linear mixing rule and the Bingham–Heitmiller model in the case of the immiscible mixture at 230 °C.

Considering the mixture of PP_1_ and PP_2_ (see Figure 11), the shear rate-based calculation method with the linear model yields the best results. This deviates the least from the measured viscosity data. For all other models, the shear stress-based calculation method is more accurate compared to the shear rate-based calculation method due to the smaller averaged deviation. Nevertheless, the shear rate-based calculation of the linear model delivers the best results.

In summary, based on the results shown, it can be recommended to apply the shear rate-based calculated linear mixing rule for homogeneous mixtures.

Looking at the PP_1_–PA12 blends, or rather the effects of the compatibilizer used, various points need to be addressed. First, in this study, the PPgMAH influences the viscosity of the pure PP slightly, as can be seen in Figure 7 and Figure 8. Other authors [21,22,23] found a strong effect of the compatibilizer on the viscosity of immiscible polymer blends. Even though the viscosity was just improved slightly in this study, it was clearly affected by the compatibilizer.

The measured viscosity of the uncompatibilized mixture cannot be described by any of the considered mixing models, as the values are below the values of the lower viscous material. The model of Lin [29] could probably describe this behavior.

The measured viscosity of the compatibilized mixture is between the viscosities of the pure materials and can be modeled by a mixing model with low deviations, as can be seen in Figure 9, Figure 10 and Figure 12. At 230 °C, the shear stress-based Arrhenius–Bersted mixing model delivers the lowest averaged absolute deviations, but the shear rate-based calculation of the Bingham–Heitmiller model shows only slightly higher deviations. At 250 °C, the shear stress-based calculation of the Bingham–Heitmiller mixing model delivers the lowest deviations, but the shear rate-based calculation of the Bingham–Heitmiller model also shows deviations below 5%. It can be concluded that the Bingham–Heitmiller model allows good prediction of the viscosity of a compatibilized mixture through both methods. This does make sense, as the Bingham–Heitmiller model assumes that the single phases of a blend do not intermix, but rather flow in separate layers [30], behavior that PP_1_ and PA12 may show because of the different polarity of these materials. Furthermore, the results show that the mixing rule of Arrhenius–Bersted also delivers good results when the shear stress-based calculation is applied. This also means that the mixing rules of Cragoe and Walther [45] would also lead to low deviations when they are applied in a shear stress-based calculation, as they behave similarly to Arrhenius’s mixing rule.

In summary, based on the results shown, it can be recommended to apply the shear rate-based calculated mixing rule of Bingham–Heitmiller for heterogeneous mixtures.

Kallel et al. [18] found a diminishing effect of the compatibilizer at high shear rates. As can be seen in Figure 9 and Figure 10, all mixing models predict the viscosity of compatibilized immiscible blends clearly better at low shear rates. At high shear rates, the measured viscosity of an immiscible mixture is clearly lower than predicted by the mixing rules, so the effect observed by Kallel et al. [18] is confirmed by the calculations and measurements.

As can be seen in Figure 7 and Figure 8, the viscosity of a compatibilized immiscible mixture increases only slightly when 5% to 20% more viscous material is added. This effect can be described by the Bingham–Heitmiller model with low deviations. Theoretically, the effect should be much stronger if 5% to 20% less viscous material were added, as can be seen in Figure 2. A reduction in the viscosity of an uncompatibilized mixture of immiscible material, as shown in Figure 7 and Figure 8, can occur when different materials slide against each other, as reported in several publications [30,31,56,57,58,59,60]. This effect can be counteracted by compatibilization with the PPgMAH, as seen in Figure 7 and Figure 8b.

The mixing models discussed in this paper, as well as the calculation methods presented, may be used for the predictive modeling and simulation of the functional zones of a single-screw extruder in the scope of a recycling process. Especially for the melt delay zone, melting zone, and conveying (metering) zone, the possibility of predicting the shear viscosity of mixtures is of particular interest.

## 5. Conclusions

In this study, the viscosity of polymer blends was studied for recycling modeling purposes. Different mixing rules were employed to calculate the shear rate-dependent viscosity curve of a mixture from viscosity curves of the involved pure materials. For this purpose, a shear rate-based calculation method and a newly developed stress-based calculation method were compared. The predicted mixture viscosities were compared to experimentally determined values. The polymer blends were created from two PP grades and from PP and PA12 at varying proportions, with the latter mixtures also being blended with 4 wt% compatibilizer of the PP. To assess the accuracy of the mixing rules used, the calculated viscosity was compared to data of the same blends measured with a high-pressure capillary rheometer.

The main conclusions of this paper are that for the miscible PP1–PP2 blends, several mixing rules can be applied to achieve an estimation of the viscosity with a deviation of less than 5% compared to the measured viscosity. Using the new shear stress-based calculation method, even smaller deviations can be reached. Out of the subjected mixing rules, a simple linear model, surprisingly, deviated the least from the measurements.

For the immiscible blends made from PP and PA12, a much higher average absolute deviation was calculated for most models. Only the model proposed by Bingham and Heitmiller differs less than 5% from the measured viscosity at both applied temperatures. In contrast to the miscible blends, the linear model is by far the most inaccurate.

Based on the results shown, it is recommended to apply the shear rate-based linear mixing model for mixtures of different grades from the same polymer type and the shear rate-based Bingham–Heitmiller model for compatibilized mixtures of different polymers. The shear stress-based models partially deliver better results, but they are much more complicated to apply due to the more complex evaluation and higher number of evaluation steps. The viscosity of uncompatibilized immiscible mixtures cannot be estimated by the applied mixing models, as the viscosity of the blends is partially lower than the viscosity of the lower viscous incorporated material. The proposed mixing models can be used to estimate the viscosity of mixtures, which is crucial when designing an extrusion process for recycling or for recycled materials.

## Figures and Tables

**Table 1:** Polymeric materials used, including their abbreviations for this work, the MFR/MVR, and the processing temperature, as given in the material data sheets.

Grade	Abbreviation	MFR/MVR ^1^	Processing Temperature ^1^
PP HB600TF	PP_1_	2 g/10 min (230 °C/2.16 kg)	200–260 °C
PP HD234CF	PP_2_	8 g/10 min (230 °C/2.16 kg)	N.A.
PA12 Grilamid L25	PA12	20 cm^3^/10 min (275 °C/5 kg)	230–250 °C
Orevac CA 100	PPgMAH	10 g/10 min (190 °C/0.325 kg)	Wide range

^1^ Derived from the material data sheet.

**Table 2:** Blends of PP_1_, PA12 and PPgMAH for the preliminary mechanical trials.

Base Polymer	Concentration Base Polymer (wt%)	Contaminating Polymer	Concentration Contaminating Polymer (wt%)	Coupling Agent	Concentration Coupling Agent (wt%)
PP_1_	79.2	PA12	20	PPgMAH	0.8
PP_1_	78.4	PA12	20	PPgMAH	1.6
PP_1_	77.6	PA12	20	PPgMAH	2.4
PP_1_	76.8	PA12	20	PPgMAH	3.2
PP_1_	76.0	PA12	20	PPgMAH	4.0

**Table 3:** Blends of PP_1_, PP_2_, PA12 and PPgMAH used in rheological testing.

Base Polymer	Concentration Base Polymer (wt%)	Contaminating Polymer	Concentration Contaminating Polymer (wt%)	Coupling Agent	Concentration Coupling Agent (wt%)
PP_1_	100	-	-	-	-
PP_1_	90	PP_2_	10	-	-
PP_1_	80	PP_2_	20	-	-
PP_1_	70	PP_2_	30	-	-
PP_1_	50	PP_2_	50	-	-
PP_1_	30	PP_2_	70	-	-
PP_1_	10	PP_2_	90	-	-
PP_2_	100	-	-	-	-
PP_1_	95	PA12	5	-	-
PP_1_	90	PA12	10	-	-
PP_1_	85	PA12	15	-	-
PP_1_	80	PA12	20	-	-
PP_1_	96	-	-	PPgMAH	4.00
PP_1_ *	91.20	PA12	5	PPgMAH	3.80
PP_1_ *	86.40	PA12	10	PPgMAH	3.60
PP_1_ *	81.60	PA12	15	PPgMAH	3.40
PP_1_ *	76.80	PA12	20	PPgMAH	3.20
PA12	100	-	-	-	-

* Mixtures containing PPgMAH have been labeled with their concentration of PP_1_ and PPgMAH, added together in future tables and figures.

## Data Availability

The original contributions presented in the study are included in the article, further inquiries can be directed to the corresponding author.

## Appendix A

**Table A1:** Fit parameters for the BCY model of PP_1_ and PP_2_ blends.

Mixture	Temperature in C	$\eta_{0}$ in Pa s	n	B in s	a	Deviation in %
100% PP_1_	200	32,326.24	0.08	0.22	0.28	1.35
90% PP_1_; 10% PP_2_	200	12,443.06	0.15	0.18	0.38	1.35
70% PP_1_; 30% PP_2_	200	8374.55	0.19	0.17	0.46	1.86
50% PP_1_; 50% PP_2_	200	3070.17	0.21	0.06	0.69	2.04
30% PP_1_; 70% PP_2_	200	3509.35	0.15	0.04	0.44	1.13
10% PP_1_; 90% PP_2_	200	1177.96	0.24	0.02	0.92	0.68
100% PP_2_	200	2333.07	0.06	0.01	0.41	1.82

**Table A2:** Measured viscosity data of the PP_1_ and PP_2_ blends. MTSRate, measured true shear rate; MTVisc, measured true viscosity. First part of the data.

MTSRate 100% PP₁ 0% PPgMAH 0% PA12 200 °C	MTVisc 100% PP₁ 0% PPgMAH 0% PA12 200 °C	MTSRate 90% PP₁ 0% PPgMAH 10% PA12 200 °C	MTVisc 90% PP₁ 0% PPgMAH 10% PA12 200 °C	MTSRate 70% PP₁ 0% PPgMAH 30% PA12 200 °C	MTVisc 70% PP₁ 0% PPgMAH 30% PA12 200 °C	MTSRate 50% PP₁ 0% PPgMAH 50% PA12 200 °C	MTVisc 50% PP₁ 0% PPgMAH 50% PA12 200 °C
11.16	2232.93	22.88	1371.27	11.08	2036.03	10.99	1611.33
28.65	1284.30	62.20	765.16	29.18	1167.16	25.71	1066.53
66.05	783.48	147.80	443.14	65.29	710.51	60.79	723.14
153.17	452.61	337.98	253.31	147.19	434.26	144.73	418.97
341.96	260.99	776.74	141.21	337.98	247.99	331.32	243.59
802.71	142.49	1891.64	71.79	752.22	142.51	737.86	140.87
1964.28	70.55	4404.70	36.31	1803.28	75.34	1760.32	75.57
4413.11	36.85	12,867.65	14.71	4386.00	36.87	4342.20	36.75
9667.29	19.81			9910.65	19.08	10,085.44	18.48
16,904.08	13.21			17,179.09	12.78	17,565.97	12.24

**Table A3:** Measured viscosity data of the PP_1_ and PP_2_ blends. MTSRate, measured true shear rate; MTVisc, measured true viscosity. Second part of the data.

MTSRate 30% PP₁ 0% PPgMAH 70% PA12 200 °C	MTVisc 30% PP₁ 0% PPgMAH 70% PA12 200 °C	MTSRate 10% PP₁ 0% PPgMAH 90% PA12 200 °C	MTVisc 10% PP₁ 0% PPgMAH 90% PA12 200 °C	MTSRate 0% PP₁ 0% PPgMAH 100% PA12 200 °C	MTVisc 0% PP₁ 0% PPgMAH 100% PA12 200 °C
22.30	975.10	22.31	794.66	10.51	967.74
57.64	621.41	55.52	568.03	24.67	738.95
135.50	387.95	133.94	355.43	56.13	530.42
316.94	228.79	311.27	210.94	130.53	349.94
738.55	128.72	734.70	119.15	308.89	208.92
1651.40	72.84	1700.51	64.73	736.65	116.15
3908.87	38.75	3835.44	35.59	1742.50	61.83
9402.64	19.19	9026.72	18.40	4085.47	32.17
16,691.30	12.67	16,470.85	11.85	9461.38	16.51
				16,742.36	10.91

## Appendix B

**Table A4:** Fit parameters for the BCY model of uncompatibilized and compatibilized PP_1_ and PA12 blends.

Mixture	Temperature in °C	$\eta_{0}$ in Pa s	n	B in s	a	Deviation in %
100% PP_1_	230	10,995.94	0.15	0.19	0.38	2.02
95% PP_1_; 5% PA12	230	1262.94	0.22	0.02	0.77	2.18
90% PP_1_; 10% PA12	230	1013.39	0.24	0.02	0.80	1.31
85% PP_1_; 15% PA12	230	912.107	0.27	0.02	1.00	1.61
80% PP_1_; 20% PA12	230	1026.29	0.26	0.02	1.09	1.87
96% PP_1_; 4% PPgMAH	230	3680.82	0.15	0.05	0.50	2.55
95% PP_1_–PPgMAH; 5% PA12	230	4988.348	0.15	0.06	0.44	1.86
90% PP_1_–PPgMAH; 10% PA12	230	5156.10	0.15	0.06	0.47	1.44
85% PP_1_–PPgMAH; 15% PA12	230	4746.50	0.19	0.08	0.51	1.02
80% PP_1_–PPgMAH; 20% PA12	230	4428	0.19	0.08	0.54	1.91
100% PA12	230	1614.30	0.34	0.00	1.67	2.50
100% PP_1_	250	20,863.15	0.04	0.11	0.25	2.88
95% PP_1_; 5% PA12	250	14,769.92	0.04	0.07	0.26	1.11
90% PP_1_; 10% PA12	250	14,645.04	0.04	0.07	0.26	2.26
85% PP_1_; 15% PA12	250	15,216.12	0.04	0.07	0.26	1.55
80% PP_1_; 20% PA12	250	14,560.54	0.03	0.06	0.25	1.85
96% PP_1_; 4% PPgMAH	250	12,637.34	0.08	0.08	0.27	2.67
95% PP_1_–PPgMAH; 5% PA12	250	12,496.73	0.08	0.08	0.27	2.81
90% PP_1_–PPgMAH; 10% PA12	250	11,375.75	0.07	0.07	0.28	0.85
85% PP_1_–PPgMAH; 15% PA12	250	11,765.88	0.07	0.07	0.29	1.28
80% PP_1_–PPgMAH; 20% PA12	250	12,188	0.07	0.07	0.28	1.26
100% PA12	250	2849.27	0.14	0.00	0.55	2.66

**Table A5:** Measured viscosity data of the compatibilized PP_1_ and PA12 blends at 230 °C. MTSRate, measured true shear rate; MTVisc, measured true viscosity. First part of the data.

MTSRate 96% PP₁ 4% PPgMAH 0% PA12 230 °C	MTVisc 96% PP₁ 4% PPgMAH 0% PA12 230 °C	MTSRate 91.2% PP₁ 3.8% PPgMAH 5% PA12 230 °C	MTVisc 91.2% PP₁ 3.8% PPgMAH 5% PA12 230 °C	MTSRate 86.4% PP₁ 3.6% PPgMAH 10% PA12 230 °C	MTVisc 86.4% PP₁ 3.6% PPgMAH 10% PA12 230 °C
10.38	1412.35	10.97	1564.79	10.64	1712.37
25.99	1064.44	26.95	993.20	26.36	1192.64
61.74	652.26	61.29	648.47	62.53	740.10
142.06	401.29	142.01	395.85	144.59	444.09
329.13	231.52	327.63	229.52	333.45	255.71
757.00	130.03	746.47	130.55	781.32	139.96
1762.59	70.03	1742.62	70.70	1852.57	72.75
4144.25	36.18	4183.34	35.84	4339.74	37.12
12,936.16	13.75	11,361.95	15.52	11,601.36	16.27
36,861.17	5.43	22,425.61	8.66	22,980.69	9.02

**Table A6:** Measured viscosity data of the compatibilized PP_1_ and PA12 blends at 230 °C. MTSRate, measured true shear rate; MTVisc, measured true viscosity. Second part of the data.

MTSRate 81.6% PP₁ 3.4% PPgMAH 15% PA12, 230 °C	MTVisc 81.6% PP₁ 3.4% PPgMAH 15% PA12, 230 °C	MTSRate 76.8% PP₁ 3.2% PPgMAH 20% PA12, 230 °C	MTVisc 76.8% PP₁ 3.2% PPgMAH 20% PA12, 230 °C	MTSRate 0% PP₁ 0% PPgMAH 100% PA12, 230 °C	MTVisc 0% PP₁ 0% PPgMAH 100% PA12, 230 °C
11.07	1691.22	10.99	1807.83	19.58	1585.16
26.70	1063.02	27.52	1119.55	46.85	1523.74
61.57	701.99	62.70	710.60	108.22	1261.41
143.60	418.52	142.68	435.84	251.82	899.55
330.80	242.57	332.36	250.91	603.50	565.40
770.16	134.08	773.87	137.79	1478.09	307.94
1773.18	72.34	1691.48	78.38	2957.63	195.87
4056.73	38.54	3752.81	44.31	5849.49	145.42
9610.83	19.42	12,319.38	16.63		
17,343.03	12.45	−100,932.70	4.75		

**Table A7:** Measured viscosity data of the compatibilized PP_1_ and PA12 blends at 250 °C. MTSRate, measured true shear rate; MTVisc, measured true viscosity. First part of the data.

MTSRate 96% PP₁ 4% PPgMAH 0% PA12, 250 °C	MTVisc 96% PP₁ 4% PPgMAH 0% PA12, 250 °C	MTSRate 91.2% PP₁ 3.8% PPgMAH 5% PA12, 250 °C	MTVisc 91.2% PP₁ 3.8% PPgMAH 5% PA12, 250 °C	MTSRate 86.4% PP₁ 3.6% PPgMAH 10% PA12, 250 °C	MTVisc 86.4% PP₁ 3.6% PPgMAH 10% PA12, 250 °C
10.80	1189.36	10.93	1322.41	11.02	1383.71
25.58	814.29	26.67	854.35	26.82	874.38
58.83	562.47	60.82	562.02	60.47	583.10
138.02	347.63	139.29	348.70	139.53	361.31
320.93	203.55	320.02	206.56	320.78	212.82
733.86	116.28	732.93	118.09	731.11	122.36
1676.36	64.87	1693.41	65.09	1707.39	66.82
4007.33	33.68	4024.87	33.77	4100.18	34.10
10,003.69	16.01	12,306.15	13.16	10,033.85	16.48
18,172.23	10.08	31,211.24	5.74	18,075.70	10.47

**Table A8:** Measured viscosity data of the compatibilized PP_1_ and PA12 blends at 250 °C. MTSRate, measured true shear rate; MTVisc, measured true viscosity. Second part of the data.

MTSRate 81.6% PP₁ 3.4% PPgMAH 15% PA12, 250 °C	MTVisc 81.6% PP₁ 3.4% PPgMAH 15% PA12, 250 °C	MTSRate 76.8% PP₁ 3.2% PPgMAH 20% PA12, 250 °C	MTVisc 76.8% PP₁ 3.2% PPgMAH 20% PA12, 250 °C	MTSRate 0% PP₁ 0% PPgMAH 100% PA12, 250 °C	MTVisc 0% PP₁ 0% PPgMAH 100% PA12, 250 °C
11.00	1451.30	10.96	1438.56	8.24	2148.32
26.73	923.44	26.89	917.96	17.71	1757.46
61.94	600.94	62.02	591.61	38.64	1385.98
142.24	362.84	140.62	363.69	84.95	1065.02
320.30	217.49	315.78	220.48	190.67	765.44
720.29	127.16	730.69	126.24	441.92	499.65
1718.32	68.64	1744.21	67.03	1071.40	285.17
4242.49	33.72	4248.64	33.41	2712.68	143.32
9871.78	16.98	9590.90	17.31	6487.90	71.19
17,178.96	11.33	16,460.06	11.87		
